# Supplementary material for: Identification of photoperiod-induced specific miRNAs in the adrenal glands of Sunite sheep (Ovis aries)
Source: Front Vet Sci. 2022 Jul 22;9:888207. doi: 10.3389/fvets.2022.888207 (PMC9354845; doi:10.3389/fvets.2022.888207)
Supplement: Supplementary file 1 [file Data_Sheet_1.ZIP › Supplementary file/miRDeep2 file.zip!/miRDeep2 file/Know_Structure/SP42A3/oar-mir-411a.pdf]

miRBase precursor : oar-mir-411a
Total read count : 23099
oar-miR-411a-5p read count : 21347
oar-miR-411a-3p read count : 1750
remaining reads : 2

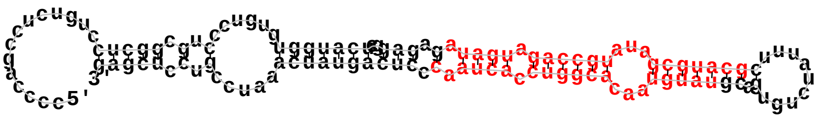

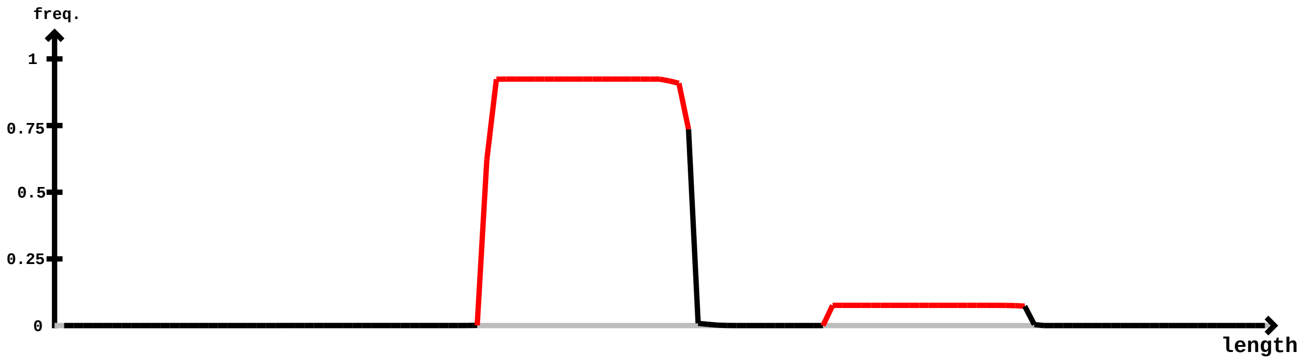

oar-miR-411a-5p

oar-miR-411a-3p

```
5'- ccccagccucuguccucggcgucccugugugguacucggagagauaguagaccguauagcguacgcuuuaucugugacguauguaacacgguccacuaacccucaguaucaaauccguccucgag -3'   exp
    ..............(((((.(.(......(((((((..(((.(.((((.((((((...((((((((.........).)))))))...)))))).)))).).)))))))))).....).).)))))   reads   mm
    ......................cccugugugguacucggagagau................................................................................   2       0
    .........................................agauaguagaccguauagcgu...............................................................   2       0
    .........................................agauaguagaccguauagcgua..............................................................   1       0
    .........................................agauaguagaccguauagcguac.............................................................   1       0
    ..........................................gauaguagaccguauagcgu...............................................................   1       0
    ..........................................gauaguagaccguauagcguac.............................................................   2       0
    ..........................................gauaguagaccguauagcguacg............................................................   9       0
    ..........................................gauaguagaccguauagcguacgc...........................................................   1       0
    ...........................................auaguagaccguauagc.................................................................   1       0
    ...........................................auaguagaccguauagcgu...............................................................   130     0
    ...........................................auaguagaccguauagcgua..............................................................   139     0
    ...........................................auaguagaccguauagcguac.............................................................   2617    0
    ...........................................auaguagaccguauagcguacg............................................................   11361   0
    ...........................................auaguagaccguauagcguacgc...........................................................   44      0
    ...........................................auaguagaccguauagcguacgcu..........................................................   36      0
    ...........................................auaguagaccguauagcguacgcuuu........................................................   2       0
    ............................................uaguagaccguauagcgu...............................................................   24      0
    ............................................uaguagaccguauagcgua..............................................................   62      0
    ............................................uaguagaccguauagcguac.............................................................   1363    0
    ............................................uaguagaccguauagcguacg............................................................   5434    0
    ............................................uaguagaccguauagcguacgc...........................................................   42      0
    ............................................uaguagaccguauagcguacgcu..........................................................   32      0
    ............................................uaguagaccguauagcguacgcuu.........................................................   29      0
    ............................................uaguagaccguauagcguacgcuuu........................................................   5       0
    .............................................aguagaccguauagcguacg............................................................   5       0
    .............................................aguagaccguauagcguacgcu..........................................................   4       0
    ...............................................................................uauguaacacgguccacu............................   5       0
    ...............................................................................uauguaacacgguccacua...........................   18      0
    ...............................................................................uauguaacacgguccacuaa..........................   45      0
    ...............................................................................uauguaacacgguccacuaac.........................   1595    0
    ...............................................................................uauguaacacgguccacuaacc........................   85      0
    ...............................................................................uauguaacacgguccacuaaccc.......................   1       0
    ................................................................................auguaacacgguccacuaac.........................   1       0
```
